# Modification and Functionalization of Zeolites for Curcumin Uptake

**DOI:** 10.3390/ma15186316

**Published:** 2022-09-12

**Authors:** Ewelina Musielak, Agnieszka Feliczak-Guzik, Mietek Jaroniec, Izabela Nowak

**Affiliations:** 1 Faculty of Chemistry, Adam Mickiewicz University, Uniwersytetu Poznańskiego 8, 61-614 Poznań, Poland; 2 Department of Chemistry and Biochemistry, Kent State University, Kent, OH 44242, USA

**Keywords:** hierarchical zeolites, FAU-type zeolite, biologically active substances, curcumin, piperine

## Abstract

This work shows that hierarchical zeolites are promising systems for the delivery of biologically relevant hydrophobic substances, such as curcumin. The validity of using piperine as a promoter of curcumin adsorption was also evaluated. The use of pure curcumin is not medically applicable due to its low bioavailability and poor water solubility. To improve the undesirable properties of curcumin, special carriers are used to overcome these shortcomings. Hierarchical zeolites possessing secondary mesoporosity are used as pharmaceutical carrier systems for encapsulating active substances with low water solubility. This porosity facilitates access of larger reagent molecules to the active sites of the material, preserving desirable adsorption properties, acidity, and crystallinity of zeolites. In this work, methods are proposed to synthesize hierarchical zeolites based on a commercial FAU-type zeolite. Studies on the application and adsorption kinetics of curcumin using commercial FAU-type zeolite and hierarchical zeolites based on commercial FAU-type zeolite are also included.

## 1. Introduction

Biotechnology and biomedical science are currently experiencing rapid development, especially in the delivery of active substances into the human body [1,2,3]. Despite the wide range of these substances, the greatest challenge of modern science has been their delivery to a specific site in the body and their release for the desired time. Therefore, more attention is paid to the search for suitable carriers of active substances allowing a controlled release of drugs with poor water solubility and their transfer to a specific cell or tissue [4]. According to the existing literature, liposomes, phospholipids [5,6,7], lipid nanoparticles [8], carbon, polymeric or porous materials, such as zeolites [9,10], are used as drug carriers so far.

The use of zeolites as matrices for the incorporation of therapeutic substances permits their sustained release due to the unique properties of zeolites [11]. These materials are characterized by high biocompatibility with various types of cells and tissues [12] and are used successfully as carriers of various therapeutic substances [13,14,15,16,17,18,19,20,21]. Moreover, they are used in veterinary medicine to improve the morphology and productivity of the bacterial flora of the gastrointestinal tract of animals to release metabolically essential ions, to improve the nutritional status of mammals, to increase the level of immunity, and to remove toxic products of digestion [22]. The desire to use zeolites as drug-delivery systems has been dictated by the low cost of their manufacturing, the wide range of available zeolite types, and their wide availability [23]. However, a major problem in using some conventional natural microporous zeolites occurring in the form of small nanoparticles as carriers of active substances is their cytotoxicity (e.g., erionite can cause mesothelioma and gastric cancer) [24].

A new class of materials that may be an alternative to microporous zeolites are hierarchical zeolites. These materials, in addition to micropores (primary pores with sizes <2 nm according to the IUPAC classification [25]), possess secondary porosity, such as mesopores (pore sizes from 2 nm to 50 nm) and/or small macropores (pore sizes above 50 nm). These materials show some applications, e.g., in catalysis [26], adsorption, or medicine [27] as drug carriers allowing the delayed release of active ingredients and their transport to the desired tissues and organs [28]. In addition, hierarchical zeolites play an important role in regulating micronutrient levels in the body and have been successfully used in cancer diagnosis and therapy. Recent studies show that these materials, with appropriate particle size, provide an excellent mechanism for transporting active substances into the human body [12,29,30,31].

Based on recent trends, it is expected that any carriers and therapeutic substances used should be as natural as possible. Therefore, finding active substances with the highest possible efficacy and low toxicity is important. Among natural substances with therapeutic activity, those of plant origin stand out. A particularly interesting substance is curcumin, which is a natural polyphenol of plant origin extracted from the root of *Curcuma longa*. Curcumin is a β-diketone, which exists exclusively in a cyclic enol form stabilized by hydrogen bonding. The entire structure of curcumin consists of three rings connected by ethylene groups. Since curcumin is characterized by high geometric anisotropy, the appropriately substituted curcumin analogs (the group of curcumin “B” analogs) can be expected to exhibit liquid crystalline properties. Curcumin is widely known and used worldwide in many different forms. In Japan it is added to tea, in India curcumin is present in spice curry, in China it is used as a coloring agent, in Korea it is served in beverages, and in Pakistan, it is used as an anti-inflammatory agent [32]. The properties of curcumin have been known for thousands of years. In the Far East, it was used in traditional medicine. Today, *Curcuma longa* has been used in Asian countries as a medicinal herb due to its potential antioxidant, anti-inflammatory [33], antimutagenic, antimicrobial [34,35], and anticancer properties [36,37]. Curcumin supplements show a myriad of therapeutic benefits, but most of them are due to their antioxidant and anti-inflammatory effects [32]. Currently, curcumin is used in traditional medicines for various diseases, including wound healing, urinary tract infections, and liver diseases. Considerable research efforts have been devoted to the study of its chemopreventive and antiproliferative effects. Despite its potential benefits, one disadvantage of curcumin in the human body is its poor bioavailability [32], which is mainly due to its low absorption rate and rapid metabolism. These “inadequacies” of curcumin can be eliminated using various additives to block its metabolic pathway and improve its bioavailability. One of the best examples of a curcumin-enhancing substance is piperine, a known bioavailability enhancer, which is the main active ingredient in black pepper [38] and causes a 2000% increase in curcumin bioavailability [39].

Based on the literature review, there are many available curcumin carriers, primarily based on nanoparticles, that can be used for its effective delivery to specific sites in the body. These include biological carriers (e.g., exosomes), protein-based biopolymers [40], polysaccharide biopolymers [40,41,42], and silica nanoparticles [43]. The latter enables the delivery of active ingredients that are poorly water soluble, have minimal targeting ability, and have a low therapeutic index. In 2020, Guo et al. [44] reported the synthesis of a nanoplatform using pH-sensitive ferritin nanocages co-loaded with the curcumin and liquid fluorocarbon, perfluorohexane (PFH), inside the core and conjugated with tumor-targeting molecule folic acid (FA) outside the shell, referred to as multifunctional nanoplatform (FA-FCP). These materials demonstrated high biocompatibility in in vitro and in vivo studies. Furthermore, FA-FCP exhibited an excellent tumor-targeting and acoustic/pH-triggered curcumin release.

Recently, Khatun et al. [45] described the effect of montmorillonite (MMT) and genipin on the release of curcumin from genipin-crosslinked chitosan/MMT nanoparticles prepared by the ionic gelation method. The release of curcumin from the nanoparticles increased with decreasing pH of the medium, MMT, and genipin content.

In contrast, Kim et al. [46] prepared meso-macrostructured silica materials containing curcumin by mineralizing dispersions of solid lipid nanoparticles in micellar solutions.

Punf et al. [47] reported the synthesis of poly-(DL-lactide-co-glycolide) (PLGA) nanoparticles (Cur-NPs) in the presence of the modified stabilizer, Pluronic F127, using a nanostructuring technique. On the surface of Cur-NPs, the carboxyl end group of the modified Pluronic F127 was conjugated to amino terminus of anti-P-glycoprotein (P-gp) (Cur-NPs-APgp). The results indicated that Cur-NPs-APgp targeted P-gp on the surface membrane of KB-V1 cells, thereby enhancing the cellular uptake and cytotoxicity of curcumin.

Moreover, a satisfactory therapeutic effect was observed after using nanorange formulations of curcumin. The synthesis of nanocurcumin-based formulations was carried out by two most effective methods: ionic gelation and antisolvent precipitation. Most of these formulations, however, remain at the proof-of-concept stage, and experiments have only been conducted on preclinical models; thus, the lack of knowledge about the risks associated with curcumin nanoformulation in humans is a major concern [48].

To the best of our knowledge, hierarchical zeolites have not been used so far as curcumin carriers. Hence, the aim of this study is to present a method of incorporating curcumin in a hierarchical zeolite obtained from one of the popular commercial zeolites, FAU, using ionic and non-ionic structuring agents. The optimization of the deposition process of this active substance onto the selected carrier enabled the development of an efficient procedure for preparing this hierarchical zeolite loaded with curcumin. The effects of solvent, temperature, and promoter addition on the curcumin adsorption process are also investigated.

## 2. Materials and Methods

### 2.1. Materials

Commercial FAU zeolite (SiO_2_/Al_2_O_3_ = 5.1) was purchased from Alfa Aesar (Kandel, Germany). Structuring agents: ionic CTABr (hexadecyltrimethylammonium bromide), nonionic: Lutrol F127 (Pluronic F127), Brij S10 (polyethylene glycol octadecyl ether), and silicon source—tetraethyl orthosilicate (TEOS), were purchased from Sigma-Aldrich Sp. z.o.o (Poznań, Poland). Ammonia aqueous (solution below 25%) was purchased from P.P.H. “STANLAB” Sp. z o.o. (Lublin, Poland). Curcumin (purity > 90%) was purchased from ROTH, Poland. The piperine was purchased from TriMen Chemicals SA (Lodz, Poland). All other chemicals and solvents were of analytical purity and were used without further purification.

### 2.2. Methodology

#### Synthesis of Hierarchical Zeolites

**Scheme 1:**
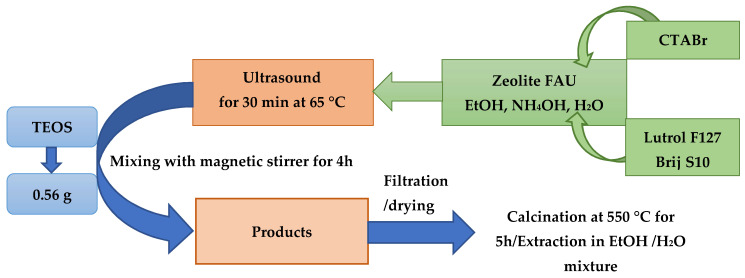
Illustration of the synthesis of hierarchical zeolites.

Hierarchical zeolites were synthesized from commercial FAU zeolite. FAU zeolite (0.50 g), ethanol (96%, 60.00 g), ammonia aqueous solution (1.25 g), structuring agents, such as CTABr, Lutrol F127, or Brij S10 (0.35 g), and distilled water (100.00 g) were weighed in a bottle made of polypropylene (PP), respectively. The whole mixture was ultrasonicated at 65 °C for 30 min. Next, the mixture was transferred to a magnetic stirrer with a heating function, where 0.56 g of TEOS was added under continuous stirring. The whole mixture was stirred for 4 h at 65 °C. After the specified time the mixture was removed from the magnetic stirrer and washed by gravity on a glass funnel with a mixture of distilled water and ethanol (1:1). The precipitate after washing was left to dry for 24 h (Scheme 1) [49].

The next step of the synthesis was calcination of the resulting zeolite material to remove the structuring agent: the process was carried out at 550 °C for 5 h, and the temperature rise time was 2.5 h (heating rate was 3.5 °C/min) [49].

### 2.3. Characterization of Hierarchical Materials

The obtained hierarchical zeolites and those loaded with curcumin were subjected to physicochemical characterization using the following techniques: X-ray diffraction (XRD), low-temperature nitrogen adsorption/desorption, differential scanning calorimetry (DSC), elemental analysis, Fourier transform infrared spectroscopy (FT-IR), transmission electron microscopy (TEM), and scanning electron microscopy (SEM).

#### 2.3.1. X-Ray Diffraction

X-ray diffractograms of hierarchical zeolites without and with curcumin were obtained using a Bruker D8 ADVANCE powder diffractometer with Johansson monochromator, with CuK_α_ radiation (λ = 0.15406 nm). The materials were analyzed in the following ranges: small angles (SAXD: 2ϴ = 0.6–0.8°) with a scan rate of 0.02°/3 s and wide angles (WAXD: 2ϴ = 6.0–60.0°) with a scan rate of 0.05°/1 s. Unit cell parameters for zeolite-type crystal structures were calculated from the XRD data using Rietveld refinement.

#### 2.3.2. Low-Temperature Nitrogen Adsorption/Desorption Isotherms

Measurements of nitrogen adsorption/desorption isotherms on micro- and mesoporous zeolite materials were performed using the ASAP 2420 MICROMERITICS apparatus. The samples were degassed under vacuum at 120 °C for 24 h before adsorption measurements. Isotherms were obtained at ca. −196 °C in the relative pressure range p/p_0_ from 0.02 to 0.99. The specific surface area of the materials was determined using the BET (Brunauer–Emmett–Teller) method. In turn, the pore size distributions were calculated using the KJS (Kruk–Jaroniec–Sayari) method based on the BJH (Barrett–Joyner–Halenda) algorithm [50]. The micropore volume and mesopore volume were established using the t-plot method.

#### 2.3.3. Differential Scanning Calorimetry

The thermal properties of the materials studied were determined using a DSC 8500 differential scanning calorimeter (Perkin Elmer, Waltham, MA, USA). The instrument was calibrated before measurements. The samples were sealed in aluminum containers and then gradually heated from 0 °C to 300 °C in a flow of nitrogen (20 mL/min) at a rate of 5 °C per minute.

#### 2.3.4. Elemental Analysis

Elemental composition determinations of the synthesized curcumin carriers were performed on a FLASH 2000, THERMO SCIENTIFIC instrument. The Organic Elemental Analysis apparatus uses the method of dynamic full combustion of samples in a reduction-oxidation furnace with electronically controlled temperature.

#### 2.3.5. Fourier Transform Infrared Spectroscopy

Characterization of functional groups was performed using a Cary 630 FT-IR spectrometer from Agilent Technologies equipped with an attenuated total reflection (ATR) element (ZnSe crystal). The samples were analyzed at room temperature in the wavenumber range of 4000–650 cm^−1^, with a resolution of 16 cm^−1^. For each measurement, 72 scans were performed for the sample spectrum and an additional eight background scans.

#### 2.3.6. Transmission Electron Microscopy

Transmission electron microscope was used to observe the fine structure of the materials, which cannot be distinguished by an ordinary microscope. Microscopic images of the pure zeolite material were also taken. JEOL’s Jem 1200 Ex II Electron Microscope (JEOL, Tokyo, Japan), a transmission electron microscope, was used for the study. The samples were bombarded with an electron beam of 80 kV energy.

#### 2.3.7. Scanning Electron Microscopy

The microstructures and morphology of pure and curcumin-loaded hierarchical zeolites were analyzed by scanning electron microscopy (SEM, Quanta FEG 250 (FEI)) under a low-pressure vacuum (70 Pa) and 10 kV beam accelerating voltage. The samples were placed on a carbon-coated 400 mesh copper grid to obtain SEM images.

### 2.4. Optimization of Curcumin Deposition Conditions on Commercial Zeolite of FAU Type

In the first stage of work, the process of optimizing the curcumin deposition conditions was carried out on a commercial zeolite of FAU type, which was the base for obtaining the desired structure of the hierarchical zeolite. During the optimization process, the following modifications were made: the type of solvent (acetone, ethanol, acetone/ethanol mixture), the amount of active substance applied, e.g., curcumin (50 mg, 100 mg, 150 mg), stirring time (12 h, 24 h, 36 h, 48 h), amount of piperine (0.25 mg, 0.50 mg, 1.00 mg, 2.00 mg), and drying temperature of the final product (RT, 70 °C). The exact optimization of the curcumin loading to the commercial zeolite/hierarchical zeolite is described in Section 2.4.1.

#### 2.4.1. The Process of Curcumin Loading to an FAU-Type Commercial Zeolite

After numerous experiments, an efficient method for curcumin loading to FAU-type commercial zeolite was developed and then applied to hierarchical zeolites. The process was as follows: 250.00 mg of FAU-type commercial zeolite was added to 5.00 mL of the selected solvent or solvent mixture, then the appropriate amount of curcumin was added (see Table S1) and placed in a dark bottle. The resulting solutions were stirred for 12 h, 24 h, 36 h, or 48 h. Next, the resulting mixtures were filtered, and the products were dried either in the air at RT or in an oven at 70 °C for 24 h to remove residual solvent.

Materials were denoted as follows: Y/R/C/P, where Y stands for the type of commercial zeolite, R for the type of solvent used (M: ethanol/acetone mixture; A: acetone, E: ethanol), C—the amount of loaded curcumin, P—the amount of piperine added.

The standard curcumin solution (concentration 0.01 mg/mL) to determine the characteristic wavelength corresponding to the maximum absorbance of curcumin was scanned using a UV-Vis Carry 50 spectrophotometer in the range of 200–800 nm. The characteristic wavelength corresponding to the maximum absorbance of curcumin in each solvent is 425 nm.

When the conditions for loading curcumin to a commercial FAU zeolite were optimized, it was possible to select the most favorable conditions to conduct the process and apply for obtaining curcumin-loaded hierarchical zeolites. Acetone proved to be the best solvent, the most favorable amount of curcumin was 150.00 mg, and the amount of piperine was 0.25 mg. The optimum stirring time was 24 h, and the material after filtration was most preferably air dried at room temperature.

Accordingly, the process of curcumin loading to the hierarchical zeolite was carried out as follows: 250.00 mg of hierarchical zeolite were combined with 5.00 mL of acetone. Then, 150.00 mg of curcumin was added to the mixture and placed under magnetic stirring, where the active ingredient was applied at room temperature within 24 h. After this time, the resulting mixtures were filtered, and the products were air dried at room temperature for 24 h to remove residual solvent (Table 1). The materials were designated as follows FAU/Z/C/P, where FAU stands for the type of commercial zeolite, Z for the type of structuring agent, C refers to the amount of curcumin added, and P refers to the amount of piperine.

#### 2.4.2. Determination of the Percentage of Curcumin Loaded into Porous Material

To determine the percentage of curcumin loaded to the selected carrier (% load), it is necessary to know the exact weight of the active substance (curcumin) used in the preparation of the mixture containing it with the selected solvent and the final weight of the product with the active substance. The efficiency of the application of curcumin to the selected carrier was determined using Equation (1).

(1) $$\%\;load=\frac{final\;mass\;of\;curcumin\;fixed\;in\;the\;carrier\;\left[g\right]}{mass\;of\;the\;mixture\;\left(curcumin+carrier\right)\left[g\right]}\times 100\%$$

Equation (1): Formula to calculate the percentage of curcumin loading to the carrier [40].

#### 2.4.3. pH_ZPC_ Analysis

To determine the pH_ZPC_ of each sample, 0.03 g of adsorbent was added to 10 mL of sodium chloride (NaCl 0.01 mol L^−1^), and the pH of the solution was adjusted to the required pH in the range of 2–10 with hydrochloric acid (HCl 0.1 mol L^−1^) and/or sodium hydroxide (0.1 mol L^−1^). The resulting mixture was stirred using a magnetic stirrer at 200 rpm for 24 h at room temperature (25 °C). A pH-meter from the Mettler Toledo company was used to determine pH_ZPC_ [51].

#### 2.4.4. Langmuir and Freundlich Adsorption Isotherms

Langmuir’s adsorption isotherm is applicable for monolayer adsorption, according to which the adsorbed quantity corresponds to the number of the occupied surface sites. The linear form of the Langmuir isotherm equation can be expressed as (Equation (2)):

(2) $$\frac{C_{e}}{q_{e}}=\frac{1}{K_{L}\times q_{max}}+\frac{C_{e}}{q_{max}}$$

Equation (2): the linear form of the Langmuir isotherm equation [52]. Where *C_e_* is the equilibrium concentration [mg/mL], *q_e_* is the equilibrium adsorption amount [mg/g], *K_L_* is the apparent Langmuir adsorption equilibrium constant corresponding to the units used, and *q_max_* is the the maximum adsorption capacity of the adsorbent [mg/g].

In addition, the Langmuir isotherm model can be characterized using the dimensionless separation coefficient, *R_L_* (Equation (3)):

(3) $$R_{L}=\frac{1}{K_{L}\times C_{0}}$$

where *C*_0_ is the initial concentration [mg/mL].

Equation (3): dimensionless separation coefficient is *R_L_* [52].

The value of *R_L_* can be used to determine whether adsorption is unfavorable (*R_L_* > 1), favorable (0 < *R_L_* < 1), or linearly favorable (*R_L_* = 1).

The Freundlich isotherm is an empirical isotherm, which assumes that the adsorbent surface is heterogeneous. This model is described by Equation (4).

(4) $$log\;q_{e}=log\;K_{F}+\frac{1}{n}\;log\;C_{e}$$

Equation (4): the Freundlich isotherm [52] where *q_e_* is the amount of curcumin adsorbed at equilibrium concentration [mg/g], *C_e_* is the equilibrium concentration of curcumin [mg/ mL], *K_F_* is the Freundlich constant], and 1/n is the heterogeneity Freundlich parameter. Moreover, the slope 1/n ranging between 0 and 1 is a measure of surface heterogeneity, which is more heterogeneous as its value gets closer to zero [52].

## 3. Results and Discussion

### 3.1. Characterization of the Hierarchical Zeolites without and with Curcumin

#### 3.1.1. X-Ray Diffraction Studies

One of the main methods for the identification of nanometer-sized materials, such as self-organized structures, is XRD. The method is mainly used to determine the structure of the sample studied and observe phase transitions and catalytic reactions occurring on the surface.

**Figure 1:**
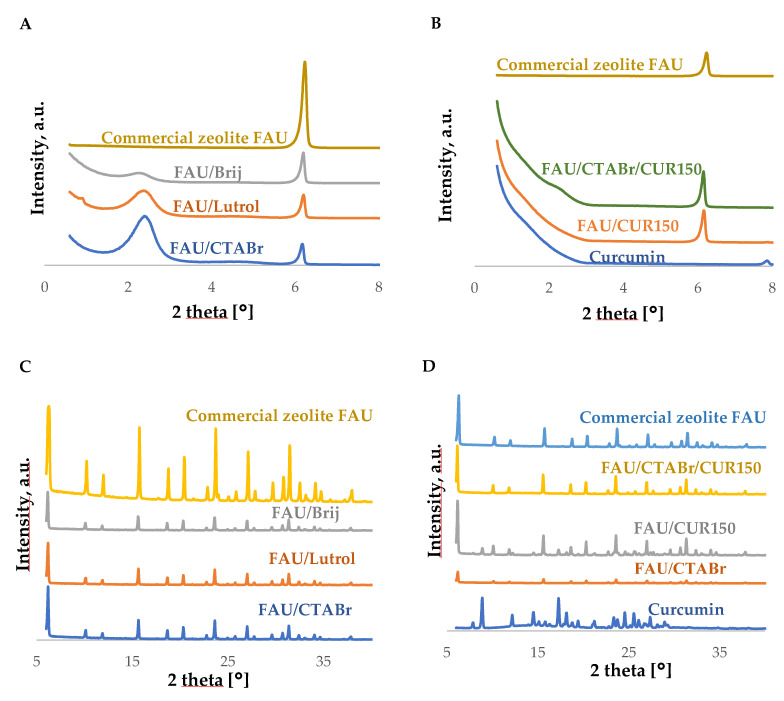
Small-angle (**A**,**B**) and wide-angle (**C**,**D**) diffractograms of hierarchical materials based on FAU-type commercial zeolite before and after curcumin loading along with spectra of the FAU-type commercial zeolite and the active substance standard.

In all diffractograms taken in the low-angle range for hierarchical zeolites without curcumin (Figure 1A), an intense and broad reflection is visible at an angle of 2θ~2.5°, possibly reflecting an additional mesoporous structure in hierarchical materials. This reflection is not observed on the pattern of the commercial material.

As one can see from the calculation of unit cell parameters presented in Table 2, the cell increased slightly after the treatment with surfactants. The smallest increase was observed for the sample modified with Lutrol. After loading with curcumin, the unit cell parameter further increased (data not presented here).

The XRD pattern of FAU zeolite loaded with curcumin (Figure 1B) shows one small reflection in the low-angle range at an angle of 2θ, about 7.5°~8.0°, referring to pure curcumin. This reflection is not observed for zeolite materials with the active substance applied, which may indicate that the active substance is incorporated within the synthesized carriers [41]. In addition, two distinct sharp reflections are observed in the wide-angle range of the diffractogram for pure curcumin at angles of 2θ~9.0° and 17.4°, which are not observed in the diffractograms of materials with curcumin. Similarly, to the diffractogram obtained in the low-angle range, this may indicate the incorporation of curcumin into the hierarchical zeolite structure (Figure 1C,D) [41].

On the contrary, the diffractograms of the curcumin-loaded materials show a small reflection in the 2θ~2.5° range, confirming the preservation of the additional mesoporous structure of the hierarchical materials (Figure 1B) [41].

The wide-angle diffractograms (Figure 1C) of all curcumin-loaded samples are like the diffractogram of thr commercial FAU-type zeolite. No changes are visible in the characteristic reflections on the diffractograms of the hierarchical zeolites with loaded curcumin, indicating that the structure of the resulting materials is not destroyed during the loading process of the active substance.

After preliminary analysis of the samples by X-ray diffraction, the materials with the best performance were selected for further characterization, namely, hierarchical zeolite obtained with the help of the CTABr ionic surfactant and loaded with 150.00 mg of curcumin.

#### 3.1.2. Nitrogen Adsorption/Desorption Studies

The textural properties of the synthesized hierarchical materials based on commercial FAU-type zeolite, such as the specific surface area, the total pore volume, the micropore volume, the mesopore volume, the average pore size and the pore distribution, were evaluated based on the nitrogen adsorption/desorption isotherm data.

**Figure 2:**
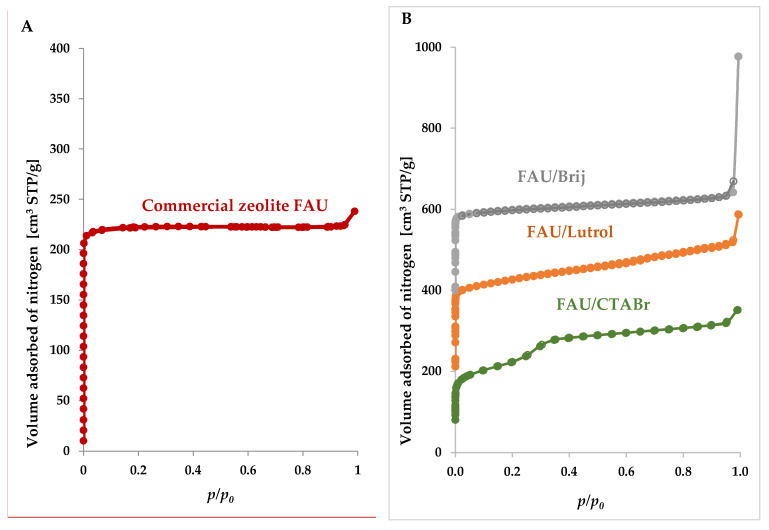
Nitrogen adsorption (filled circles)/desorption (open circles) isotherms for (**A**) FAU-type commercial zeolite and (**B**) hierarchical zeolites derived from FAU-type commercial zeolite. In panel B the top and middle isotherms are shifted by 400 and 200 cm^3^/g STP, respectively.

Nitrogen adsorption/desorption isotherm measured for a commercial FAU-type zeolite (Figure 2A and enlarged isotherms are presented in Figure S1) shows type I according to IUPAC (International Union of Pure and Applied Chemistry) classification, which is characteristic of microporous materials [42]. In the case of hierarchical materials synthesized with the addition of surfactants and TEOS, the overall isotherms are a combination of type I and IVb, the latter according to the IUPAC classification refers to mesoporous materials (Figure 2B) [42]. The incremental rise in the relative pressure at *p*/*p*_0_~0.35, typical for mesoporous materials, was especially evidenced for a material derived from the cationic surfactant, CTABr (Figure 2B). The low-pressure step can be observed in microporous materials at very low relative pressures, *p*/*p*_0_ < 0.05.

The nitrogen adsorption/desorption isotherms were obtained to confirm the presence of both micropores associated with the crystal structure of zeolites (primary porosity) and mesopores (secondary porosity) formed during the modification process. For the materials studied in the high relative pressure region, a rapid increase in the volume of nitrogen adsorbed can also be observed, which indicates the formation of secondary porosity called textural [43,44].

The specific surface area of the materials was determined using the BET (Brunauer–Emmett–Teller) method and the S_BET_ ranges from 588 to 892 m^2^/g. The specific surface area of S_BET_ is the highest for the hierarchical zeolite obtained with the help of CTABr. On the other hand, the materials obtained with the help of the nonionic surfactant, Brij S10, showed the lowest specific surface area compared to the starting material (commercial zeolite). As can be seen from Table 2, the specific surface area, S_BET_, determined for the hierarchical zeolites obtained with help of CTABr is significantly higher compared to those prepared with the help of non-ionic surfactants (Lutrol F127, Brij S10). Hierarchical zeolite prepared with the help of ionic surfactant (CTABr) is characterized by a large volume of mesopores (a marked increase from 0.05 to 0.32 cm^3^/g is observed). The average pore size of all the samples studied is similar and is in the range of 3.10–3.40 [42].

#### 3.1.3. Differential Scanning Colorimetry Studies

DSC is another method used to determine the physicochemical properties of the hierarchical zeolites studied. The technique consists of measuring the difference in heat flux delivered to the sample studied and reference sample. Using this technique, it is possible to measure the difference between the heat flow that occurs when the heat is absorbed or released by the sample due to thermal effects, such as melting, crystallization, chemical reactions, polymorphic transformations, or evaporation processes [45]. Heat transfer during temperature-controlled measurements provides information about the structural properties of the sample [46].

**Figure 3:**
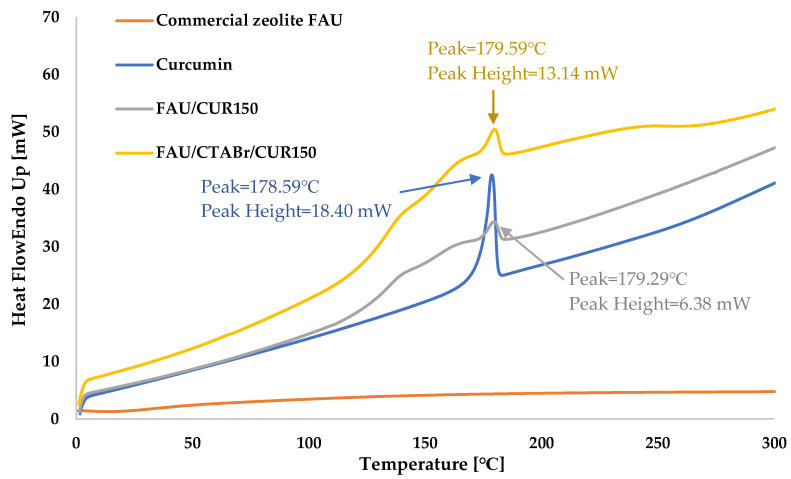
Recorded DSC thermograms for the tested materials in the temperature range from 0 °C to 300 °C.

Figure 3 shows a comparison of thermograms of hierarchical zeolite samples without and with loaded curcumin and reference samples, that is, commercial zeolite type FAU and curcumin.

DSC studies of curcumin-loaded FAU zeolite samples and pure commercial FAU zeolite were conducted in the temperature range of 0–300 °C. The commercial FAU zeolite does not show any thermal changes in the temperature range studied. On the other hand, the thermograms of pure curcumin are characterized by the presence of an exothermic peak at 178.59 °C associated with its melting (curcumin melting point 183.00 °C), which confirms previous literature data [47]. Peaks observed on the DSC thermograms of the hierarchical zeolites with curcumin are slightly shifted compared to the peak referring to pure curcumin, which may indicate the crystallization of the active ingredient in the pores of the hierarchical zeolite. According to the literature, the effect of crystallization of curcumin in the pores of the carrier may lead to a lower melting point of curcumin compared to the melting point of the same active substance outside the pores [41]. Furthermore, the shape of the peak obtained for pure curcumin at 179.29 °C (Figure 3) is sharp compared to that for the other samples (FAU/CTABr/CUR150, FAU/CUR150), which according to the literature is attributed to the melting of free curcumin located outside the pores of the material, while the peak reflecting the active substance melting in the pores is broader. This is related to the surface chemistry of the zeolite and the specific interaction of the active substance with the pore walls [41].

#### 3.1.4. Elemental Analysis Studies

Elemental analysis was performed to determine the composition of the samples studied. The results of the analysis show the nitrogen, carbon, hydrogen, and sulfur contents (wt. %) in both unmodified hierarchical zeolites and those with curcumin (Supporting Information, Table S2).

The elemental analysis confirms the effective modification of both the commercial FAU-type material and the hierarchical zeolites with curcumin, as shown in Table S2. The elemental analysis of curcumin gives 30.26 wt. %. C and 4.61 wt. % H, which can be related to the structure of the active substance containing up to 21 carbon atoms and 20 hydrogen atoms (C_21_H_20_O_6_). According to the literature data, the theoretical values of carbon and hydrogen content in curcumin are like those obtained experimentally [48].

It should be noted that after curcumin loading to both the commercial FAU type zeolite and the hierarchical zeolites, the wt. % carbon and hydrogen content increases. This confirms that there was incorporation of curcumin in the interior and onto the surface of hierarchical zeolites [48]. Analyzing the obtained results, it can be clearly stated that the highest amount of curcumin was incorporated in the case of hierarchical zeolite prepared on the basis of the ionic surfactant, CTABr. Much smaller amounts of curcumin were incorporated in the case of hierarchical zeolites synthesized using non-ionic surfactants, such as Lutrol F127 and Brij S10. Such results are related to the specific surface area and volume of the mesopores formed. By using the nitrogen adsorption/desorption technique, we know that the hierarchical zeolites obtained on the basis of CTABr are characterized by the most favorable parameters and the largest specific surface area and large volume of mesopores formed.

#### 3.1.5. Fourier Transform Infrared Spectroscopy Studies

**Figure 4:**
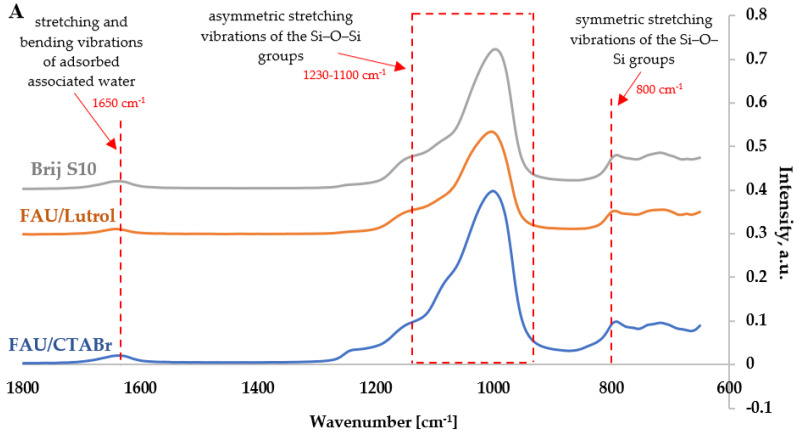

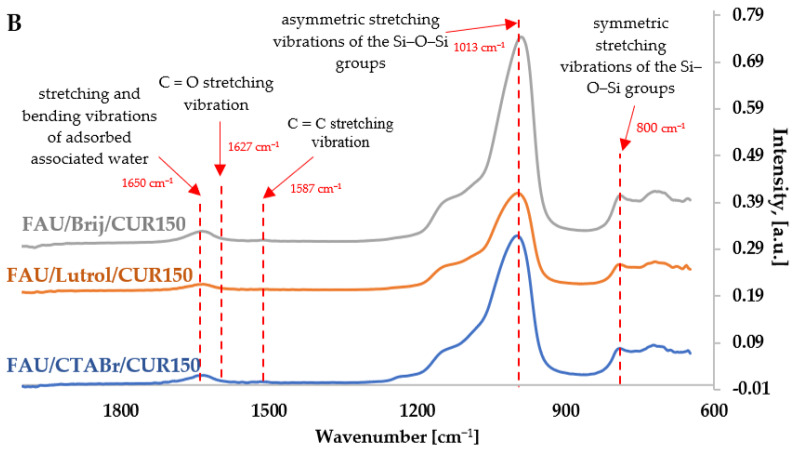
FT-IR spectra of hierarchical materials (**A**) based on FAU-type commercial zeolite and (**B**) those modified with curcumin.

FT-IR infrared spectra were recorded in the full spectroscopic range to evaluate the structure of the materials studied. FT-IR spectroscopy was used to check interactions at the molecular level between the active substance (curcumin) and the carrier (hierarchical zeolite). Figure 4A shows the FT-IR spectra in the range from 2000 to 600 cm ^−1^ of pure hierarchical zeolites obtained with the help of ionic and non-ionic surfactants, while Figure 4B summarizes the spectra of curcumin and hierarchical zeolites modified with this active ingredient.

It is observed that all the synthesized hierarchical zeolites based on commercial FAU-type zeolite are characterized by the occurrence of similar absorption bands characteristic for commercial FAU-type zeolites (Figure 4A). The spectra obtained have characteristic bands at:

- about 1230 cm^−1^ and 1100 cm^−1^, which may indicate the presence of valence stretching vibrations for asymmetric Si-O-Si(Al) bonds; the band at ca. 1100 cm^−1^,
- slightly shifted toward higher wavenumbers, indicates an increase in the elemental cell size of the modified hierarchical zeolites,
- about 800 cm^−1^, which may originate from valence stretching vibrations for symmetric Si-O-Si bonds,
- approximately 1650 cm^−1^, which may indicate the presence of bending vibrations of physically adsorbed water [53,54].

Evidence for the interaction between curcumin molecules and the FAU zeolite framework was investigated by Fourier transform infrared (FT-IR) spectroscopy. As shown in Figure 4A, characteristic FT-IR bands corresponding to the lattice vibrations are observed for the pure FAU zeolite sample in the range from 1700 to 800 cm^−1^. The broad band at 1013 cm^−1^ is due to asymmetric stretching vibrations of O-Si-O groups, while the band attributed to O-H deformation vibrations was recorded at 1640 cm^−1^ [55,56,57].

In the case of hierarchical zeolite with curcumin, bands attributed to the stretching vibrations of the phenolic O-H CUR bond are more pronounced than for the respective parent materials (without curcumin) on the FT-IR spectrum at 3600 cm^−1^ and 3500 cm^−1^, respectively (Figure S2). The bands at 1618 cm^−1^ and 1587 cm^−1^ are attributed to the stretching vibrations of C=O and C=C, respectively, and the band associated with the C-O-C bond is observed at approximately 1100 cm^−1^. The interaction of curcumin with the hierarchical surface of zeolite is evidenced by the presence of characteristic curcumin-derived bands that changed from 1506 to 1512 cm^−1^, from 1438 to 1442 cm^−1^, and from 1274 to 1281 cm^−1^ because of the formation of hydrogen bonds between the carrier hydroxyl group and the two phenolic hydroxyl groups, the enolic C-OH, and the methoxyl groups of curcumin [48].

#### 3.1.6. Transmission Electron Microscopy Studies

TEM provides important information about the structure of the materials studied that cannot be obtained by other techniques. In the case of zeolites, the method supplements the results of small angle X-ray analysis (XRD).

The study shows that hierarchical zeolites based on the commercial zeolite type FAU have an ordered crystalline FAU-type structure, which is characteristic of this type of material. Therefore, based on the obtained images, it can be concluded that the analysis performed by the XRD method is correct and that the hierarchical zeolites studied retain their ordered structure regardless of the surfactant used.

In the images shown in Figure S3, one can see that the particles of hierarchical zeolites closely adhere to each other. TEM images for all materials are very similar. According to the literature, the commercial FAU type zeolite contains octahedral type crystals [58]. A similar crystal shape was observed in all synthesized materials.

#### 3.1.7. Scanning Electron Microscopy Studies

**Figure 5:**
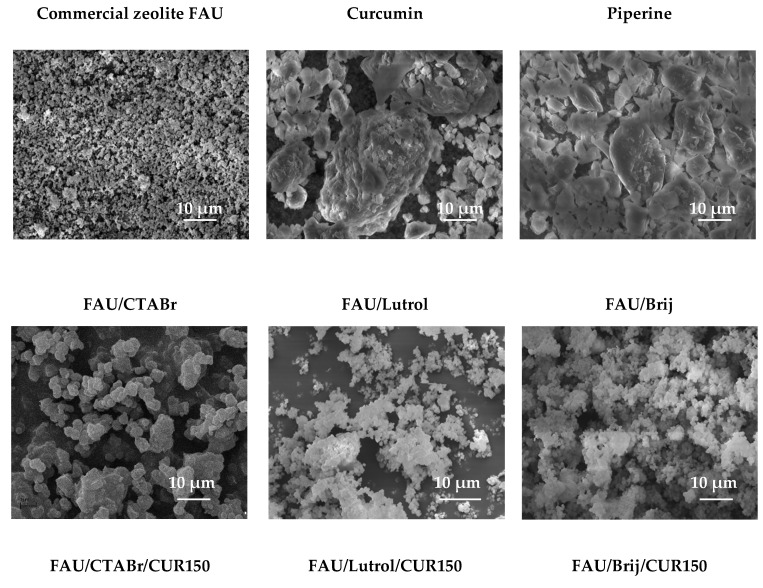

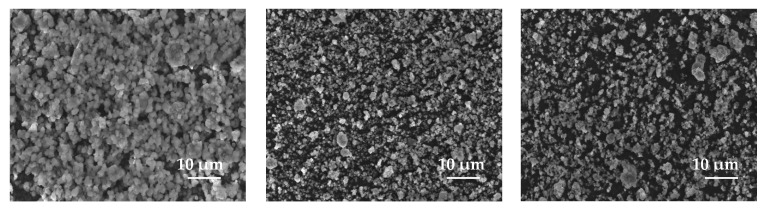
SEM images of the materials studied (10 μm scale bar).

Scanning electron microscopy was used to determine the size and morphology of the materials studied with a scale bar of 10 μm (see Figure 5).

The SEM image of pure curcumin shows irregularity of particles, while that of piperine presents more regular and smooth particles that are smaller than in the case of curcumin.

Images of pure zeolite materials are also displayed in Figure 5. The images show particle agglomeration and the irregular shapes of the unmodified hierarchical zeolites. These samples show morphological irregularities and visible roughness of the structure.

SEM images of the selected zeolite materials with curcumin show some improvement in the regularity of shape and surface of the particles studied. A smaller agglomeration of the modified hierarchical zeolites is also evident. In conclusion, no significant changes in the particle size are observed, indicating that curcumin can adsorb on the zeolite surface or occupy the voids inside the material structure, which is consistent with the literature data [59,60,61,62,63]. For all zeolite materials studied, the visible imperfections may be a consequence of prolonged exposure of the samples to a high vacuum and intense electron beam.

### 3.2. Adsorption of Curcumin

Table 3 shows Langmuir and Freundlich parameters of curcumin adsorption isotherms on commercial FAU zeolite and hierarchical materials.

The sorption capacity values for the materials studied are similar. They range from about 98 to about 102 (see Table 3).

**Figure 6:**
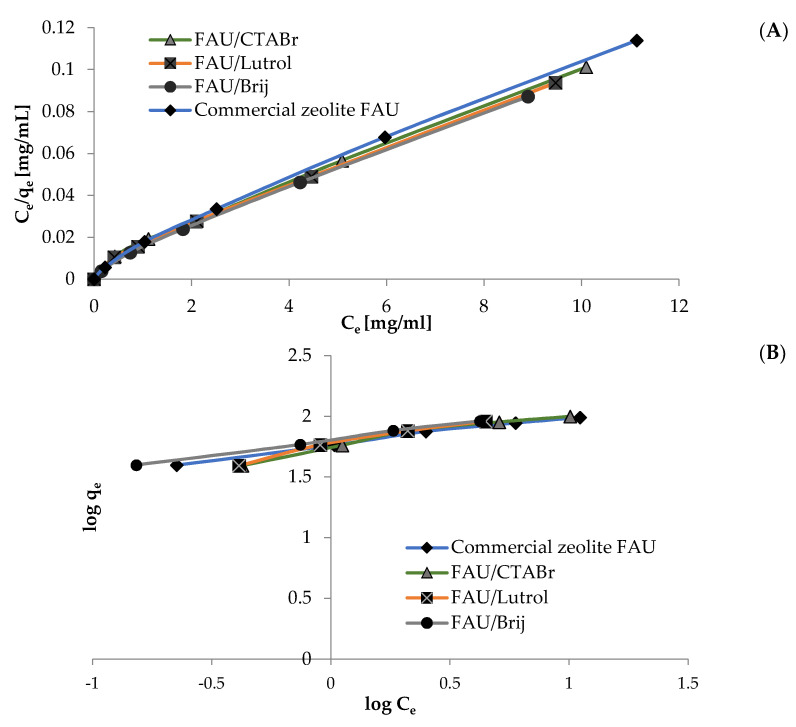
Adsorption of curcumin onto commercial zeolite and hierarchical zeolite: (**A**) Langmuir isotherm plots and (**B**) Freundlich isotherm plots (adsorbent mass = 250 mg, initial solution concentration of curcumine = 10–60 mg/mL, volume of solution = 5 mL, temperature = 22 ± 1 °C).

Information about the adsorption mechanism was obtained by comparing experimental data with the Langmuir and Freundlich adsorption isotherms. The values of q_max_ and *K_L_* are obtained from the intercept and slope of the linear plot of *C_e_*/*q_e_* versus *C_e_* (Figure 6A). Additionally, the values of *K_F_* and 1/n are obtained from the slope and the intercept of the plot of log q_e_ versus log *C_e_* (Figure 6B). A comparison of all the results is shown in Table 3. The data show that the values of the parameter 1/n are less than 1, while the value of the parameter R_L_ varies from 0.278 to 0.821, indicating that the adsorption of curcumin on the materials in question is favorable. The surface is more heterogeneous when the value of 1/*n* approaches zero [52]. Additionally, *K_F_* values are larger for hierarchical zeolites, which is related to the adsorption capacity enhancement due to the introduction of internal mesopores.

The *R*^2^ correlation coefficients for the Langmuir model are close to 1 and higher than those obtained assuming the Freundlich model, which means that the Langmuir model better represents the mechanism of curcumin adsorption on the tested samples.

### 3.3. Determination of the Percentage of Curcumin Loading to FAU-Type Commercial Zeolite and Hierarchical Zeolites

The curcumin loading into FAU-type commercial zeolite (Table 4) and hierarchical zeolites (Table 5) was calculated using Equation (1). All attempts to incorporate curcumin into hierarchical zeolites were performed three times.

The results of the analysis of curcumin loading into zeolite carriers show that the loading of the active substance increases with the increasing amount of curcumin available. The most favorable curcumin loading percentage (about 89.60%) was obtained for the FAU CTABr/CUR150 material. Although the piperine modified the structure, it did not increase the loading percentage of the active substance itself. Therefore, it can be concluded that piperine only increases the bioavailability of curcumin itself and does not influence how it is applied to carriers [64]. Thus, there is no promoting effect of piperine on the adsorption of curcumin, probable due to the steric effect playing a more important role than, for example, the London dispersion or electrostatic Starck interactions.

### 3.4. pH_ZPC_ Analysis Zeolite Materials

Zero charge point pH (pH_ZPC_) corresponds to the pH value at which the surface of a solid is considered neutral. It plays an important role during the sorption of ionic substances on solid surfaces from aqueous systems. The pH_ZPC_ studies are summarized in Table S3 and Figure S4 located in the supplemental information. The point of zero charge is an important factor that determines the linear range of pH sensitivity and then indicates the type of surface-active centers and the adsorption ability of the surface.

These measurements were used to determine the pH at which the surface charge of the material is zero. The results show that the surfaces of commercial zeolite FAU and FAU/Lutrol are close to neutral (pH_ZPC_ = 6.5), while the surfaces of FAU/CTABr and FAU/Brij are slightly acidic (pH_ZPC_ = 6.0 and 6.2, respectively). At pH values below pH_ZPC_, the surface of the test material is positively charged, while at pH values above pH_ZPC_, the surface is negatively charged [65].

### 3.5. Comparison of the Tested Materials with Other Curcumin Carriers

Various carriers can be used to stabilize the active substance; however, porous materials are less studied due to problems with the solubility of curcumin. One can imagine the use of a solid carrier with hydroxyl groups on the surface to increase the water solubility. To this end, zeolite 5A, the calcium-exchanged form of zeolite Linde type A, with a nominal pore diameter of 0.5 nm was applied, however with a low curcumin-loading capacity (only 51%) [41]. Additionally, solid lipid nanoparticles that possess solid lipid matrices can also be interesting carriers for curcumin (71.45% loading) [66]. It is noteworthy that the preparation of hierarchical zeolites is less expensive than other types of adsorbents, e.g., organically modified mesoporous materials, which makes hierarchical zeolites promising adsorbents for curcumin. Additionally, their curcumin load is the highest among all materials studied up to date.

## 4. Summary and Conclusions

The aim of this work was to study the incorporation of curcumin into hierarchical zeolites based on commercial FAU zeolite. Curcumin is a popular plant-derived therapeutic agent, which due its numerous limitations, requires the development of suitable methods for its delivery into the human body. Therefore, the current research is focused on improving its properties. The results show that the loading of curcumin into hierarchical zeolites based on commercial FAU-type zeolite is enhanced by modifying the zeolite carrier itself. Hierarchical zeolites prepared based on FAU zeolite and the ionic surfactant, CTABr, were found to be the best curcumin carriers. This observation was confirmed by DSC and XRD analyses, which showed the presence of curcumin in the pores of hierarchical zeolites and confirmed a stronger interaction of the zeolite with the active substance. FT-IR analysis detected the presence of curcumin in the zeolite structure and its binding to the carrier. Both SEM and XRD studies showed a stable structure of the hierarchical zeolite, the structural integrity of which was preserved after curcumin loading. These results confirm the presence of curcumin in the FAU zeolite carrier and support the potential use of this porous material as a carrier for active substances, most notably curcumin.

## Figures and Tables

**Table 1:** Optimization of experimental conditions for incorporating curcumin into hierarchical zeolite.

Name	Curcumin [mg]	Piperine [mg]
**FAU/CTABr/CUR50**	50.00	-
**FAU/CTABr/CUR150**	150.00	-
**FAU/Lutrol/CUR50**	50.00	-
**FAU/Lutrol/CUR150**	150.00	-
**FAU/Brij/CUR50**	50.00	-
**FAU/Brij/CUR150**	150.00	-
**FAU/CTABr/CUR50/PIP0.25**	50.00	0.25
**FAU/CTABr/CUR150/PIP0.25**	150.00	0.25
**FAU/Lutrol/CUR50/PIP0.25**	50.00	0.25
**FAU/Lutrol/CUR150/PIP0.25**	150.00	0.25
**FAU/Brij/CUR50/PIP0.25**	50.00	0.25
**FAU/Brij/CUR150/PIP0.25**	150.00	0.25

**Table 2:** Selected textural properties of hierarchical materials based on commercial zeolite type FAU (*a*_0_—unit-cell size assessed from XRD, $a_{0}=\sqrt{\frac{\left(h^{2\;}+k^{2}+l^{2}\right)\times \lambda^{2\;}}{4\;sin^{2}\;\theta }}$).

Materials	*a*_0_, [nm]	BET Surface Area [m^2^/g] S_BET_	Pore Volume [cm^3^/g]			Average Mesopore Size [nm]
			Total Pore Volume	Volume of Micropores	Mesopore Volume	
**Commercial zeolite FAU**	2.467	718	0.37	0.30	0.05	-
**FAU/CTABr**	2.474	892	0.49	0.19	0.30	3.40
**FAU/Lutrol**	2.469	696	0.46	0.19	0.27	3.10
**FAU/Brij**	2.479	588	0.43	0.17	0.26	3.20

**Table 3:** Langmuir and Freundlich parameters of the adsorption isotherms of curcumin onto commercial zeolite FAU and hierarchical materials.

Material	*q_e_* [mg/g]	Langmuir				Freundlich		
		*R* ^2^	*q_max_* [mg/g]	*K_L_*	*R_L_*	*R* ^2^	*K_F_*	1/*n*
Commercial zeolite FAU	97.93	0.993	98.04	0.027	0.2784–0.7851	0.962	57.40	0.24
FAU/CTABr	99.80	0.990	102.04	0.022	0.4330–0.8208	0.955	54.51	0.30
FAU/Lutrol	101.06	0.990	103.09	0.023	0.4207–0.8133	0.934	56.48	0.35
FAU/Brij	102.19	0.993	103.09	0.032	0.3394–0.7550	0.928	64.02	0.25

**Table 4:** Curcumin loading into commercial FAU-type zeolite.

Name	% Load	±SD
**FAU/M/CUR50**	29.30	±0.27
**FAU/M/CUR100**	47.30	±0.70
**FAU/M/CUR150**	77.60	±0.89
**FAU/E/CUR50**	30.00	±0.21
**FAU/E/CUR100**	48.10	±0.82
**FAU/E/CUR150**	54.20	±0.43
**FAU/A/CUR50**	39.20	±0.18
**FAU/A/CUR100**	61.80	±0.65
**FAU/A/CUR150**	79.60	±0.65
**FAU/A/CUR50/PIP0.25**	25.10	±0.26
**FAU/A/CUR50/PIP0.50**	32.10	±0.36
**FAU/A/CUR50/PIP1.00**	25.40	±0.70
**FAU/A/CUR50/PIP2.00**	22.10	±0.36

**Table 5:** Curcumin loading into hierarchical zeolites.

Name	% Load	±SD
**FAU/CTABr/CUR50**	39.20	±0.40
**FAU/CTABr/CUR150**	89.60	±0.40
**FAU/Lutrol/CUR50**	32.60	±0.45
**FAU/Lutrol/CUR150**	87.90	±0.65
**FAU/Brij/CUR50**	41.50	±0.60
**FAU/Brij/CUR150**	89.60	±0.80
**FAU/CTABr/CUR50/PIP0.25**	39.50	±0.36
**FAU/CTABr/CUR150/PIP0.25**	89.40	±0.45
**FAU/Lutrol/CUR50/PIP0.25**	41.30	±0.62
**FAU/Lutrol/CUR150/PIP0.25**	81.90	±0.66
**FAU/Brij/CUR50/PIP0.25**	42.30	±0.43
**FAU/Brij /CUR150/PIP0.25**	88.80	±0.70

## Data Availability

The data presented in this study are available upon request from the authors.

## Supplementary Materials

The following supporting information can be downloaded at: https://www.mdpi.com/article/10.3390/ma15186316/s1, Table S1: Optimization of different parameters in the application process of curcumin to a commercial FAU-type zeolite. Table S2: Elemental analysis of commercial FAU zeolite, hierarchical materials obtained from its base before and after curcumin application. Table S3: Final pH vs. initial pH for commercial materials. Figure S1: Nitrogen adsorption/desorption isotherms for hierarchical zeolites: FAU/CTABr (A), FAU/Brij (B) and FAU/Lutrol (C) derived from FAU-type commercial zeolite. Adsorption and desorption points are represented by closed and open circles. Note that for mesopores with diameters below 4–5 nm both adsorption and desorption branches coincide. Figure S2: FTIR spectra in the wavenumber range from 600 cm^−1^ to 4000 cm^−1^ of hierarchical materials based on commercial FAU zeolite (panel A) and these materials modified with curcumin (panel B). The enlarged portion corresponding to the range from 3000 cm^−1^ to 3800 cm^−1^ is shown at the top of each panel. Figure S3: TEM images of synthesized hierarchical zeolites, at 200 nm magnification. Figure S4: pH_final_ vs. pH_initial_ for mixture of commercial zeolite FAU, FAU/CTABr/ FAU/Lutrol and FAU/Brij.
