# Supplementary material for: Modification and Functionalization of Zeolites for Curcumin Uptake
Source: Materials (Basel). 2022 Sep 12;15(18):6316. doi: 10.3390/ma15186316 (PMC9504848; doi:10.3390/ma15186316)

Modification and Functionalization of Zeolites for Curcumin Uptake

Ewelina Musielak [1], Agnieszka Feliczak-Guzik [1], Mietek Jaroniec [2] and Izabela Nowak [1,*]

[1] Faculty of Chemistry, Adam Mickiewicz University, Uniwersytetu Poznańskiego 8, 61-614 Poznań, Poland

[2] Department of Chemistry and Biochemistry, Kent State University, Kent, OH 44242, USA

* Correspondence: nowakiza@amu.edu.pl; Tel.: +48-618291580

Table S1. Optimization of different parameters in the application process of curcumin to a commercial FAU-type zeolite.

Material	Solvent	Amount of curcumin [mg]	Amount of piperyne [mg]	Tempe-rature [°C]	Mixing time [h]
**FAU/M/ CUR50**	Ethanol/Acetone	50.00	-	RT	24
**FAU/M/CUR100**	Ethanol/Acetone	100.00	-	RT	24
**FAU/M/CUR150**	Ethanol/Acetone	150.00	-	RT	24
**FAU/E/CUR50**	Ethanol	50.00	-	RT	24
**FAU/E/CUR100**	Ethanol	100.00	-	RT	24
**FAU/E/CUR150**	Ethanol	150.00	-	RT	24
**FAU/A/CUR50**	Acetone	50.00	-	RT	24
**FAU/A/CUR100**	Acetone	100.00	-	RT	24
**FAU/A/CUR150**	Acetone	150.00	-	RT	24
**FAU/E/CUR50/70**	Ethanol	50.00	-	70	24
**FAU/E/CUR 50/12h**	Ethanol	50.00	-	RT	12
**FAU/E/CUR 50/36h**	Ethanol	50.00	-	RT	36
**FAU/E/CUR50/48h**	Ethanol	50.00	-	RT	48
**FAU/A/CUR50/PIP0.25**	Acetone	50.00	0.25	RT	24
**FAU/A/CUR50/PIP0.50**	Acetone	50.00	0.50	RT	24
**FAU/A/CUR50/PIP1.00**	Acetone	50.00	1.00	RT	24
**FAU/A/CUR50/PIP2.00**	Acetone	50.00	2.00	RT	24

Table S2. Elemental analysis of commercial FAU zeolite, hierarchical materials obtained from its base before and after curcumin application.

Material	%N	%C	%H	%S
**Pure hierarchical zeolites together with initial microporous material**				
**Commercial zeolite FAU**	0.12	0.74	0.35	0.07
**FAU/CTABr**	0.00	0.02	2.28	0.00
**FAU/Lutrol**	0.00	0.02	3.36	0.00
**FAU/Brij**	0.00	0.03	3.30	0.00
**Hierarchical materials with applied curcumin and curcumin standard**				
**Curcumin**	0.00	30.26	4.61	0.00
**FAU/CUR150**	0.00	5.69	2.64	0.00
**FAU/CTABr/CUR150**	0.00	17.30	3.24	0.00
**FAU/Lutrol/CUR150**	0.00	3.38	2.52	0.00
**FAU/Brij/CUR150**	0.00	5.30	2.81	0.00

Table S3. Final pH vs. initial pH for commercial materials.

**Commercial zeolite FAU**		**FAU/CTABr**		**FAU/Lutrol**		**FAU/Brij**	
$pH_{initial}$	pH_{final}	$pH_{initial}$	pH_{final}	$pH_{initial}$	pH_{final}	$pH_{initial}$	pH_{final}
2.0	4.0	2.0	4.0	2.0	3.0	2.0	4.0
3.0	5.0	3.0	4.5	3.0	4.0	3.0	4.5
4.0	5.5	4.0	5.0	4.0	5.5	4.0	5.0
5.0	6.0	5.0	5.5	5.0	6.0	5.0	5.5
6.0	6.5	6.0	6.0	6.0	7.0	6.0	6.0
7.0	7.0	7.0	6.5	7.0	7.0	7.0	6.5
8.0	8.0	8.0	7.0	8.0	8.0	8.0	7.0
9.0	9.0	9.0	8.0	9.0	8.5	9.0	8.0
10.0	10.0	10.0	9.0	10.0	9.0	10.0	9.0
pH_{ZPC} ~ 6.5		pH_{ZPC} ~ 6.0		pH_{ZPC} ~ 6.5		pH_{ZPC} ~ 6.2	

Figure S1. Nitrogen adsorption/desorption isotherms for hierarchical zeolites: FAU/CTABr (A), FAU/Brij (B) and FAU/Lutrol (C) derived from FAU-type commercial zeolite. Adsorption and desorption points are represented by closed and open circles. Note that for mesopores with diameters below 4-5 nm both adsorption and desorption branches coincide.

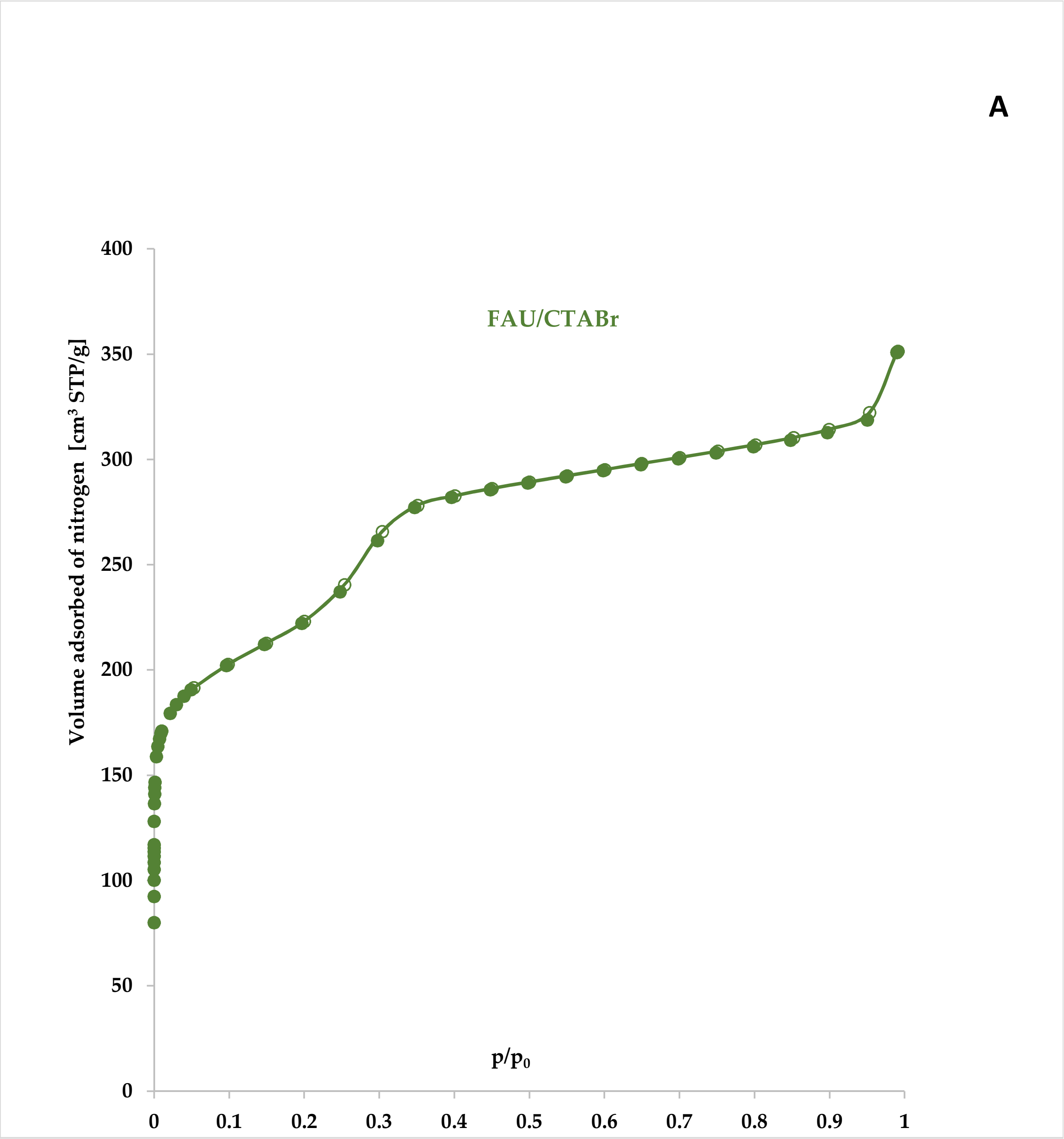

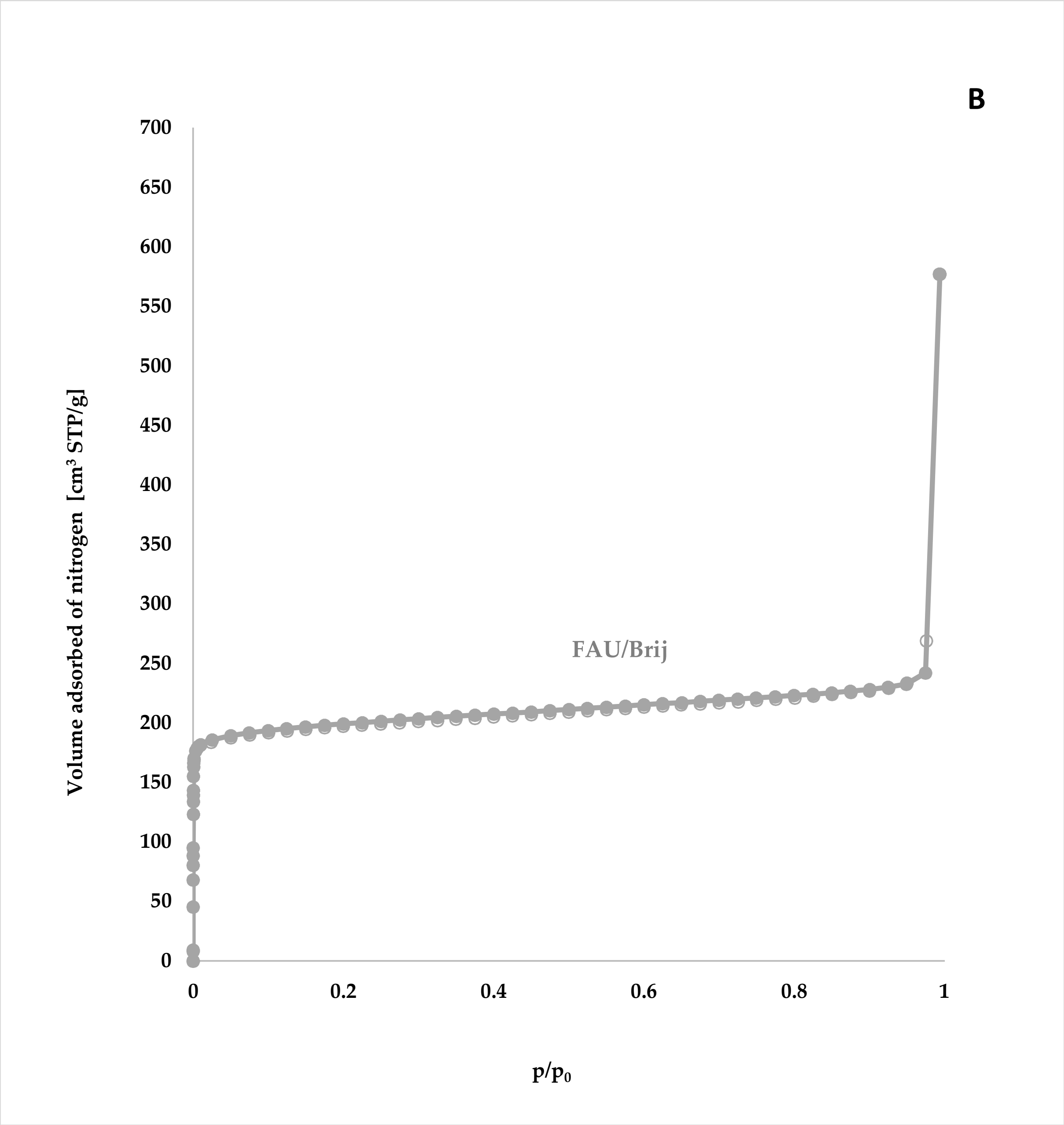
B
700
650
600
550
500
450
400
350
300
250
200
150
100
50
0
Volume adsorbed of nitrogen [cm³ STP/g]
FAU/Brij
0
0.2
0.4
0.6
0.8
1
p/p₀

C

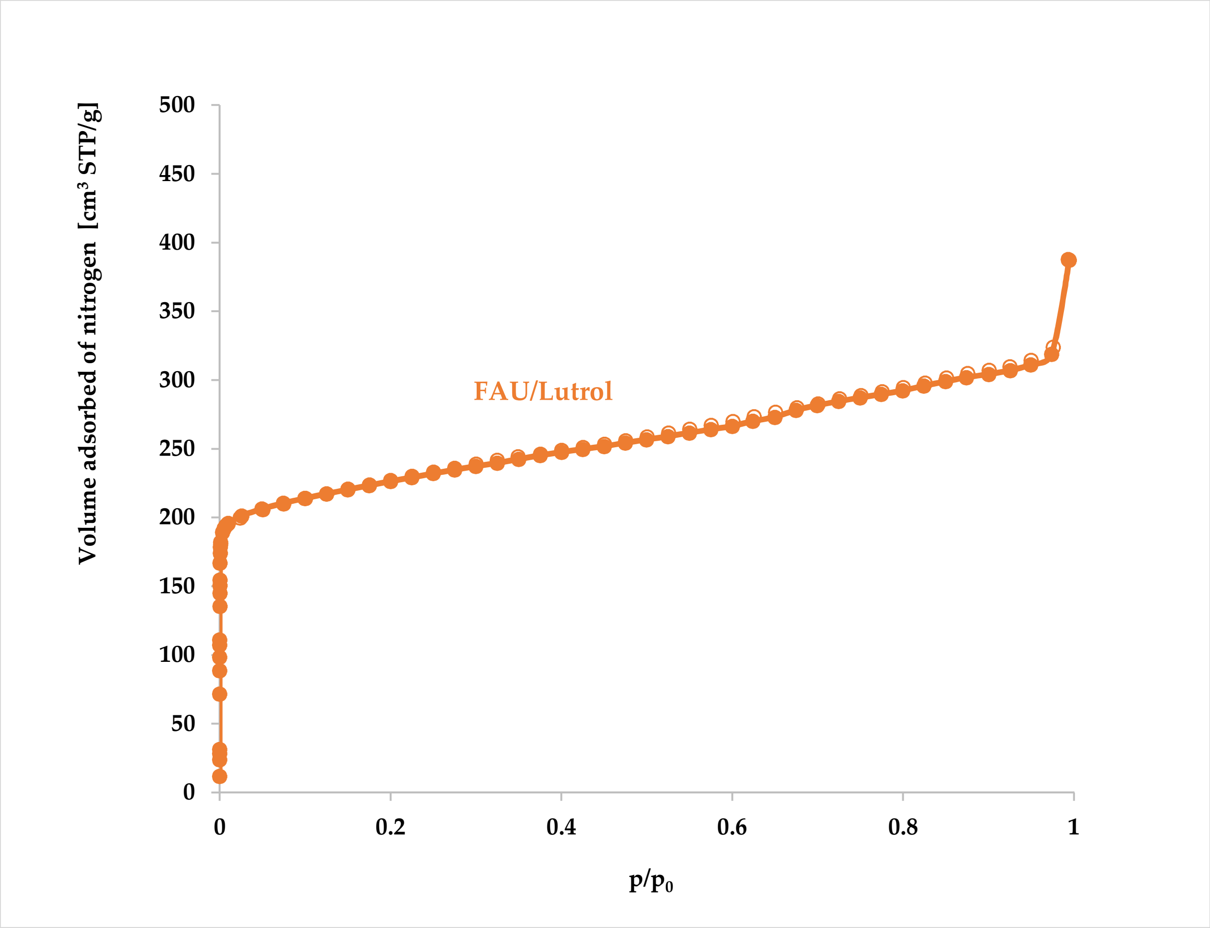

Volume adsorbed of nitrogen [cm3 STP/g]
500
450
400
350
300
250
200
150
100
50
0
0
0.2
0.4
0.6
0.8
1
p/p0
FAU/Lutrol

Figure S2. FTIR spectra in the wavenumber range from 600 cm^{-1} to 4000 cm^{-1} of hierarchical materials based on commercial FAU zeolite (panel A) and these materials modified with curcumin (panel B). The enlarged portion corresponding to the range from 3000 cm^{-1} to 3800 cm^{-1} is shown at the top of each panel.

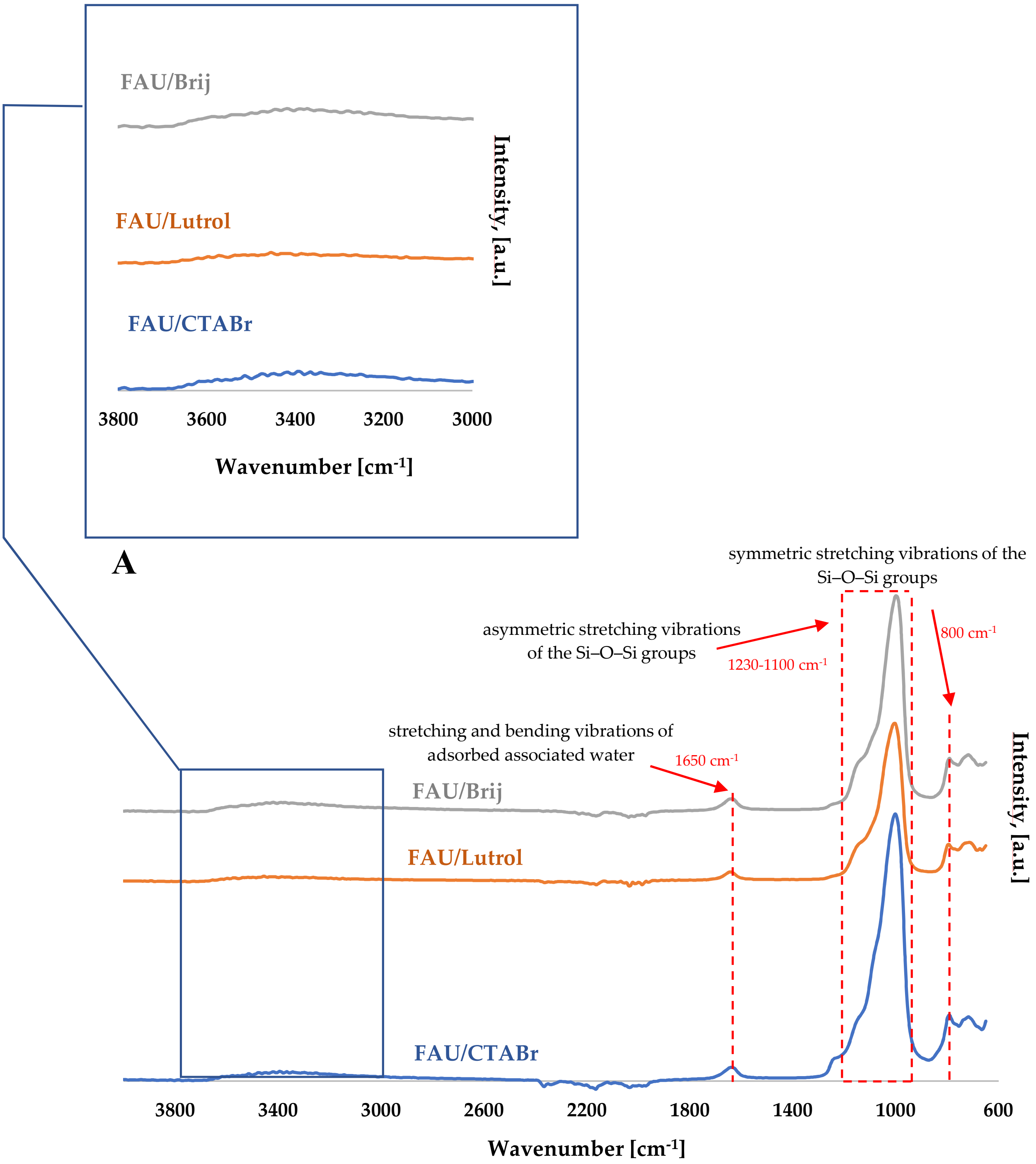

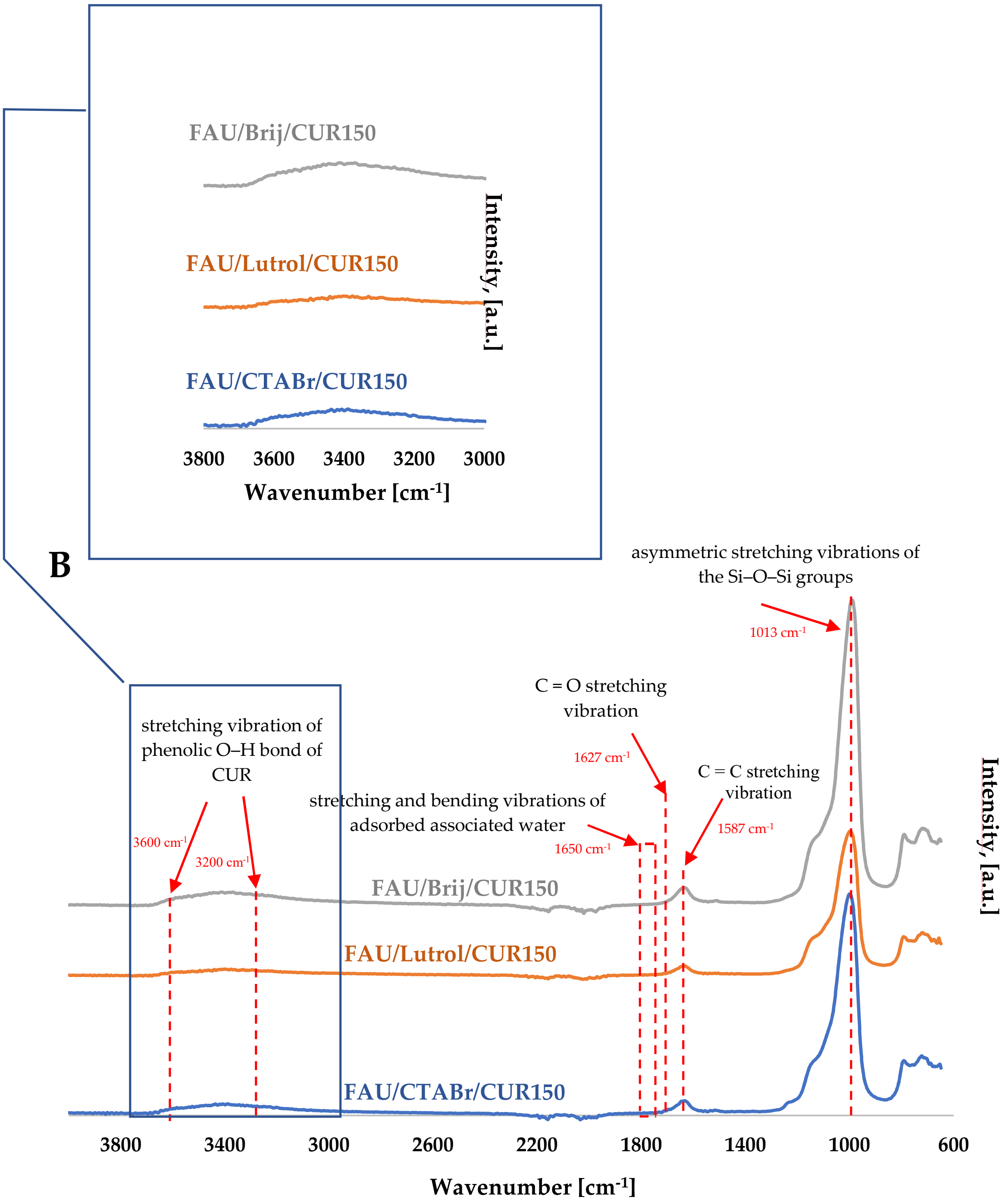

B
FAU/Brij/CUR150
FAU/Lutrol/CUR150
FAU/CTABr/CUR150
Intensity, [a.u.]
3800
3600
3400
3200
3000
Wavenumber [cm-1]
asymmetric stretching vibrations of the Si–O–Si groups
1013 cm-1
C = O stretching vibration
1627 cm-1
C = C stretching vibration
1587 cm-1
stretching vibration of phenolic O–H bond of CUR
3600 cm-1
3200 cm-1
stretching and bending vibrations of adsorbed associated water
1650 cm-1
FAU/Brij/CUR150
FAU/Lutrol/CUR150
FAU/CTABr/CUR150
Intensity, [a.u.]
3800
3400
3000
2600
2200
1800
1400
1000
600
Wavenumber [cm-1]

Figure S3. TEM images of synthesized hierarchical zeolites, at 200 nm magnification.

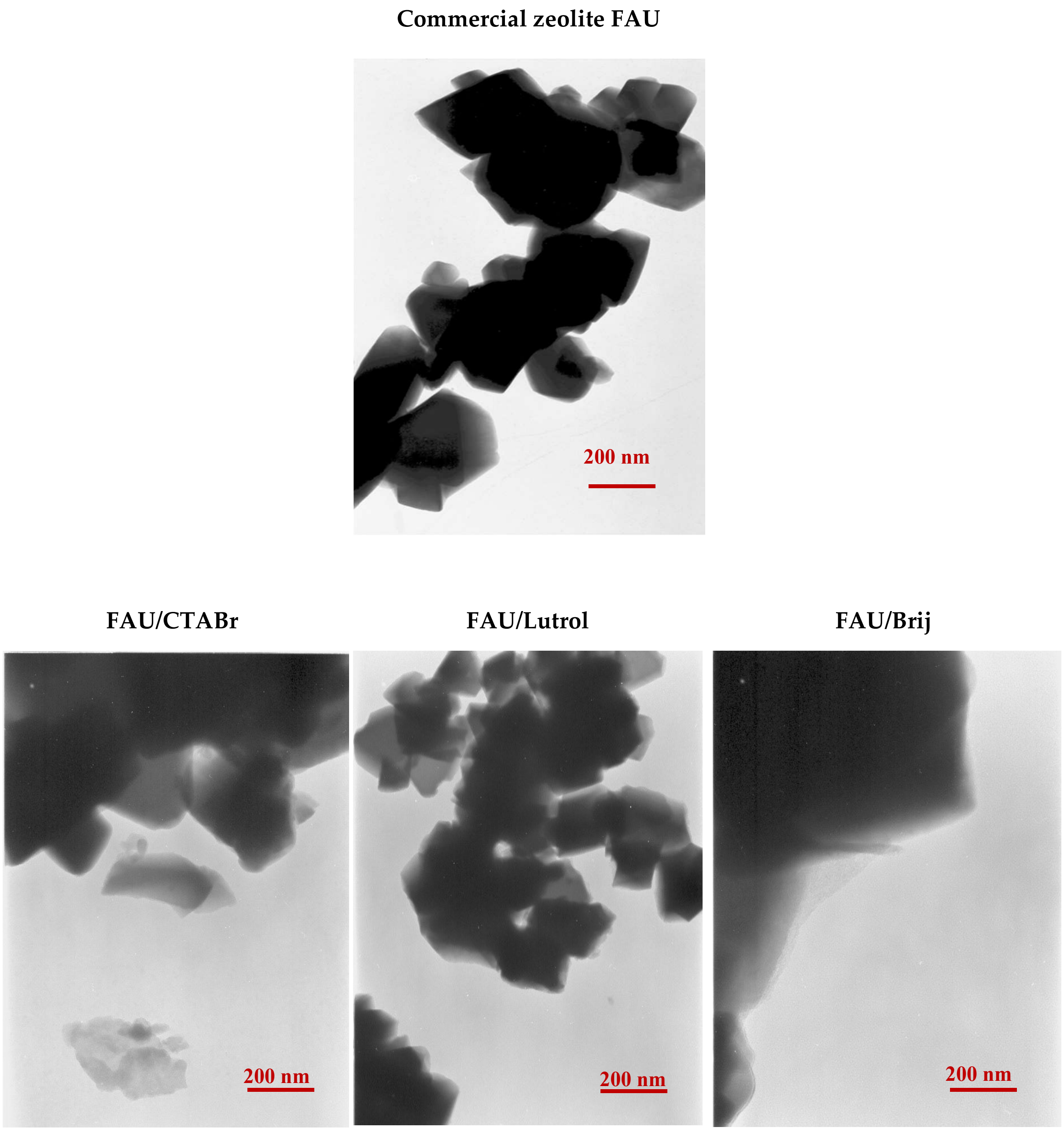

Figure S4. pH_{final} vs. $pH_{initial}$ for mixture of commercial zeolite FAU, FAU/CTABr/ FAU/Lutrol and FAU/Brij.

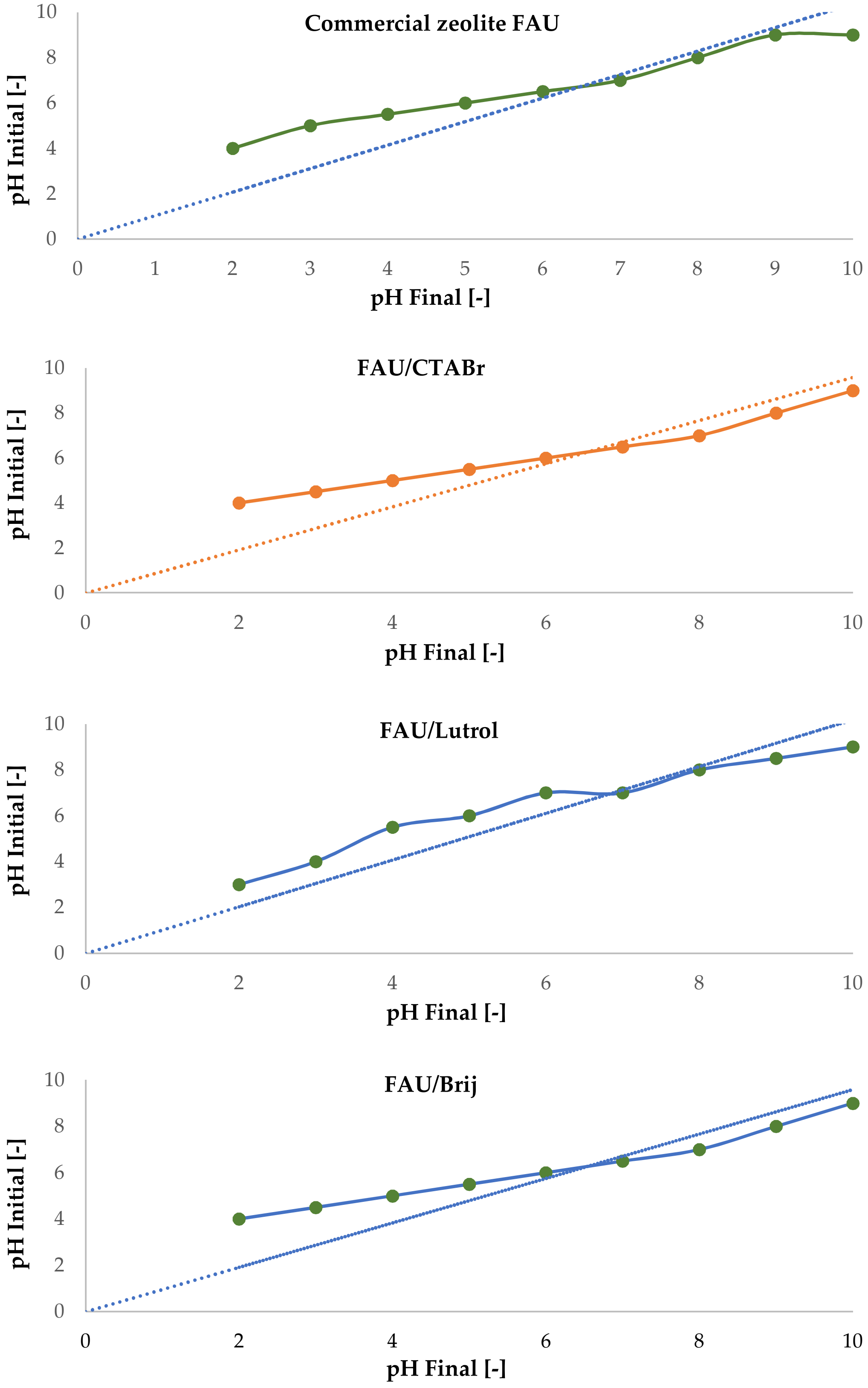

Supplement: Supplementary file 1 [file materials-15-06316-s001.zip › materials-1799759-supplementary.pdf]
